# Anticancer Potential of Piericidin A1 and Derivatives Isolated From *Streptomyces* sp. Associated With *Palythoa variabilis* From Brazilian Reefs

**DOI:** 10.1002/cbdv.202503815

**Published:** 2026-04-20

**Authors:** Bianca Del B. Sahm, Katharine G. D. Florêncio, Francisco C. L. Pinto, Ana I. V. Maia, Carlos A. M. Rocha, Paula C. Jimenez, Otília D. L. Pessoa, Tito M. C. Lotufo, Leticia V. Costa‐Lotufo, Diego V. Wilke

**Affiliations:** ^1^ Departamento De Farmacologia Universidade De São Paulo São Paulo São Paulo Brazil; ^2^ Núcleo de Pesquisa e Desenvolvimento de Medicamentos Departamento De Fisiologia e Farmacologia Universidade Federal do Ceará Fortaleza Ceará Brazil; ^3^ Departamento De Química Orgânica e Inorgânica Universidade Federal do Ceará Fortaleza Ceará Brazil; ^4^ Coordenação de Recursos Pesqueiros e Agronegócio Instituto Federal de Educação Ciência e Tecnologia do Pará Belém Pará Brazil; ^5^ Instituto Do Mar Universidade Federal de São Paulo Santos São Paulo Brazil; ^6^ Instituto Oceanográfico Universidade De São Paulo São Paulo São Paulo Brazil

**Keywords:** anticancer, holobiome, Palythoa, piericidin, zoantharians

## Abstract

Marine holobionts are an important source of bioactive natural products. Building on our previous studies of cytotoxic compounds from zoantharians of the genus *Palythoa*, we investigated the anticancer potential of bacteria associated with *Palythoa variabilis*. Ten culturable bacterial strains were isolated and screened for cytotoxic activity against metastatic prostate cancer cells (PC‐3/M) using the MTT assay. Among them, the crude extract of strain BRA‐035, identified as *Streptomyces* sp., showed the most promising bioactivity profile and was selected for cytotoxicity‐guided fractionation. This approach enabled the isolation of piericidin A1 (**1**) and the identification of two additional piericidin derivatives (**2** and **3**). piericidin A1 exhibited strong and selective cytotoxicity, with IC_50_ values ranging from picomolar to low nanomolar concentrations against ovarian (OVCAR), prostate (PC‐3 and PC‐3/M), and colorectal (HCT‐116) tumor cell lines, while remaining inactive (IC_50_ > 12 µM) against HL‐60 leukemia and B16‐F10 murine melanoma cells. Additional assays assessing cell proliferation and membrane integrity provided further insight into differential cellular sensitivity. Overall, these results demonstrate that *P. variabilis*‐associated bacteria are a valuable source of bioactive metabolites and reinforce the translational potential of piericidin A1 as a promising anticancer drug candidate.

## Graphical abstract

A *Streptomyces* spp. strain, isolated from the zoantharian *Palythoa variabilis*, produces the complex I mitochondrial inhibitor piericidin A1 with effects on cancer cells proliferation and MTT‐formazan reduction at concentrations up to 0.1 aM.

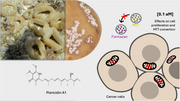

## 1 Introduction

Zoantharians (Cnidaria: Anthozoa: Hexacorallia) are soft‐bodied benthic marine animals commonly known as sea mats. They form colonies that cover rocky shores and various reef habitats worldwide and comprise a taxonomically diverse group that includes the genera *Palythoa, Zoanthus*, and *Parazoanthus* [1, 2]. Specialized metabolic pathways in zoanthids enable their survival in hostile environments and provide competitive advantages in space occupation, food acquisition, and defense against predators. These organisms are also recognized for their rich chemical arsenal, which includes notable biological activities, continuing to motivate natural product research and the development of novel pharmaceutical agents [3].

Like other cnidarians, zoantharians harbor an intrinsic microbiota that plays crucial roles in maintaining homeostasis and overall health, framing these animals as holobionts [4, 5]. In addition to these essential functions, bacterial communities associated with zoanthids are now recognized as key contributors to the production of specialized metabolites. Investigations into the biosynthetic machinery of invertebrates have revealed microbial participation in the synthesis or modification of bioactive compounds. This has led to the hypothesis that many substances previously attributed to marine invertebrates are, in fact, produced by their symbiotic or associated microorganisms [6]. Indeed, studies exploring the biosynthetic potential of marine bacteria—both associated with holobionts and free‐living—using cultivation‐dependent and independent approaches have uncovered new chemical classes, derivatives, and analogues with significant biological activity against pathogens and diseases, including cancer [7, 8, 9, 10].

Most clinically available drugs have been derived from natural sources [11]. From a sustainable development perspective, drug discovery programs focused on natural products are strategic, given their long‐standing record of yielding new medicines and, furthermore, their potential to support biodiversity conservation and equitable profit sharing. Therefore, besides being a promising fountainhead for the development of new therapeutic entities, this approach is also linked to a strong demand for the conservation of ecosystems in a climate change scenario [12]. In this context, despite their recent history compared to terrestrial counterparts, marine natural products serve as important sources of bioactive molecules [13], currently warranting 20 drugs in clinical use, mostly as antineoplastics [14].

Our group has systematically investigated bioactive molecules from marine organisms to highlight the therapeutic potential of Brazilian marine biodiversity [15]. Previously, we reported structurally diverse compounds, including palyosulfonoceramides [16] and lipidic α‐amino acids (LAAs), isolated from *Palythoa variabilis* [17]. LAAs from *P. variabilis* displayed antiproliferative activity against tumor cell lines, inducing apoptotic features and DNA fragmentation [18]. Later, two ergostane‐type sterols obtained from *P. variabilis* and *P. caribaeorum* displayed growth inhibition activity against colorectal cancer cells [19]. Chromomycin A (CA_5_), along with three new chromomycins—CA_6_, CA_7_, and CA_8_—isolated from the extracts of *Streptomyces* sp. BRA‐384 associated with *P. caribaeorum* was also identified [20] and further studied for its anticancer potential. Chromomycins are glycosylated aureolic acids that bind to the minor groove of DNA, causing double‐strand DNA damage and triggering cell stress and death [21, 22]. Our studies revealed that CA_5_ binds to the transcription factor Tbx2 and inhibits melanoma cell proliferation [22, 23].

In the present study, we screened crude extracts obtained from bacteria associated with *P. variabilis* from Northeastern Brazil for cytotoxicity and identified piericidins as the active compounds. Piericidins and derivatives are well‐known mitochondria‐decoupling cytotoxic agents [24]. The clinical use of mitochondria‐targeting compounds has attracted increasing interest in cancer therapy [25]. Still, the complex functions of this organelle and its essential role in cell survival raise concerns regarding the limited efficacy and narrow selectivity of such agents. These issues reinforce the importance of basic research to critically re‐evaluate this therapeutic strategy. Herein, the anticancer properties of piericidin A1 are discussed in light of the challenges intrinsically related to its mode of action.

## 2 Results and Discussion

### 2.1 Isolation of Bacteria Associated With *P. Variabilis* and Bioactivity Screening

**FIGURE 1:**
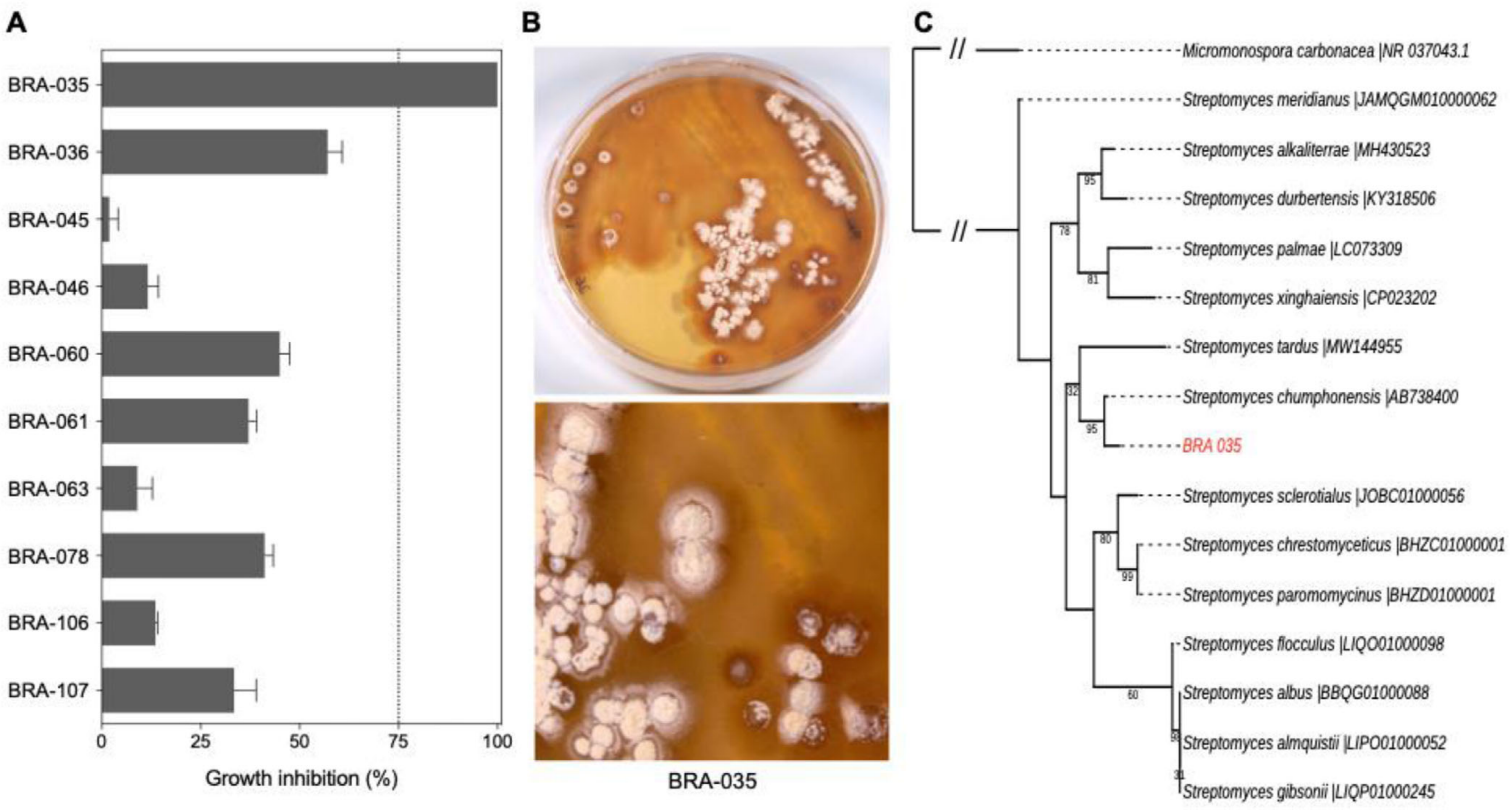
The *Streptomyces* sp. BRA‐035 strain isolated from the zoantharian *P. variabilis* exhibited the highest inhibition of tumor cell growth. (A) Bar graph showing the percentage (mean±SEM) of growth inhibition of metastatic prostate cancer cells (PC‐3/M) after 72 h incubation with crude extracts (50 µg/mL) from bacterial isolates associated with *P. variabilis*, determined by three independent experiments (*n* = 3) using the MTT assay. (B) Images of the BRA‐035 strain grown on A1 agar media. (C) Maximum likelihood phylogenetic tree based on 16S rRNA gene sequences showing the relationship between BRA‐035 and closely related *Streptomyces* species obtained from EZBioCLoud (accession codes after the |).

Initially, 10 bacterial strains associated with *P. variabilis*, collected at Taiba beach rocks, were isolated and fermented in A1 broth for 8 days under agitation (SI.1). The activity of crude ethyl acetate extracts obtained from isolated bacteria was tested on PC‐3/M human prostate carcinoma cell line. As shown in Figure 1A, the crude extract obtained from the BRA‐035 strain caused 100% growth inhibition on PC‐3/M at 50 µg/mL. The extracts obtained from other isolates were inactive against these cells. BRA‐035 colonies depict a dry, brownish appearance and produce white spores within 10 days on A1 agar medium at 28°C (Figure 1B). Molecular identification by 16S rDNA sequencing analysis suggests the BRA‐035 strain belongs to the *Streptomyces* genus (family *Streptomycetacea*; order *Actinomycetales*) and will be referred to, from now on, as *Streptomyces* sp. BRA‐035 (Figure 1C). The *Streptomyces* genus is known to be an excellent producer of metabolites with biological activity. Indeed, these bacteria are recognized as producers of a great number of clinically relevant pharmacological substances with a wide range of activities, such as antimicrobial (tetracycline, neomycin, and streptomycin), antifungal (amphotericin B, nystatin, and natamycin), anticancer (doxorubicin, daunorubicin, and mitomycin C), and anthelmintic (avermectin) [26, 27, 28, 29].

### 2.2 Bioassay‐Guided Isolation of Bioactive Compounds of *Streptomyces* sp. BRA‐035

In order to identify the bioactive substances produced by the *Streptomyces* sp. BRA‐035, 10 L of A1 culture broth was cultivated for 8 days, yielding 150 mg of crude extract. The extract was fractionated using high‐performance liquid chromatography (HPLC). The cytotoxicity of the 8 fractions obtained (SI.2) was evaluated on PC‐3/M cells by MTT assay for 72 h. As shown in Table 1, the crude extract and fractions F7 (BRA‐035‐F7) and F8 (BRA‐035‐F8) showed potent cytotoxic activity with inhibition concentration means (IC_50_) of 0.48, 47, and <16 ng/mL, respectively, against PC‐3/M cells, while the remaining fractions did not exhibit cytotoxicity (SI.3 and SI.4). HPLC analysis of fractions F7 and F8 led to the isolation and characterization ofcompound **1** (SI.5 and SI.6).

**TABLE 1:** Cytotoxicity of *Streptomyces* sp. BRA‐035 crude extract and fractions on metastatic prostate cancer cell line PC‐3/M. Results are shown as the mean inhibitory concentration (IC_50_) and 95% confidence interval (CI95%) on PC‐3/M cell line after 72 h incubation, determined by the MTT assay. Values represent the mean of three independent experiments (*n* = 3), calculated by nonlinear regression analysis using GraphPad Prism v.10.0.

Sample	IC_50_ (ng/mL)	CI95%
**BRA‐035 crude extract**	**0.048**	—^a^
BRA‐035‐F1	> 50000	—^a^
BRA‐035‐F2	> 50000	—^a^
BRA‐035‐F3	> 50000	—^a^
BRA‐035‐F4	> 50000	—^a^
BRA‐035‐F5	> 50000	—^a^
BRA‐035‐F6	> 50000	—^a^
BRA‐035‐F7	46.90	23.19–94.55
BRA‐035‐P8	< 16	‐^a^

^a^ Not determined by nonlinear regression.

**FIGURE 2:**
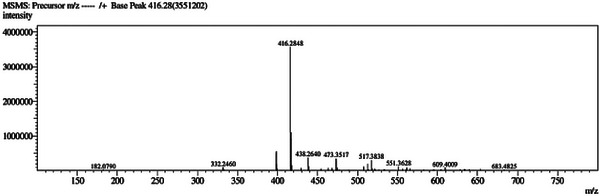
HRESIMS of pieridicin A1 (**1**). ChatGPT Plus 5.3 (accessed in February 2026) was used to enhance figure resolution. The final image was reviewed for accuracy by the authors.

**FIGURE 3:**
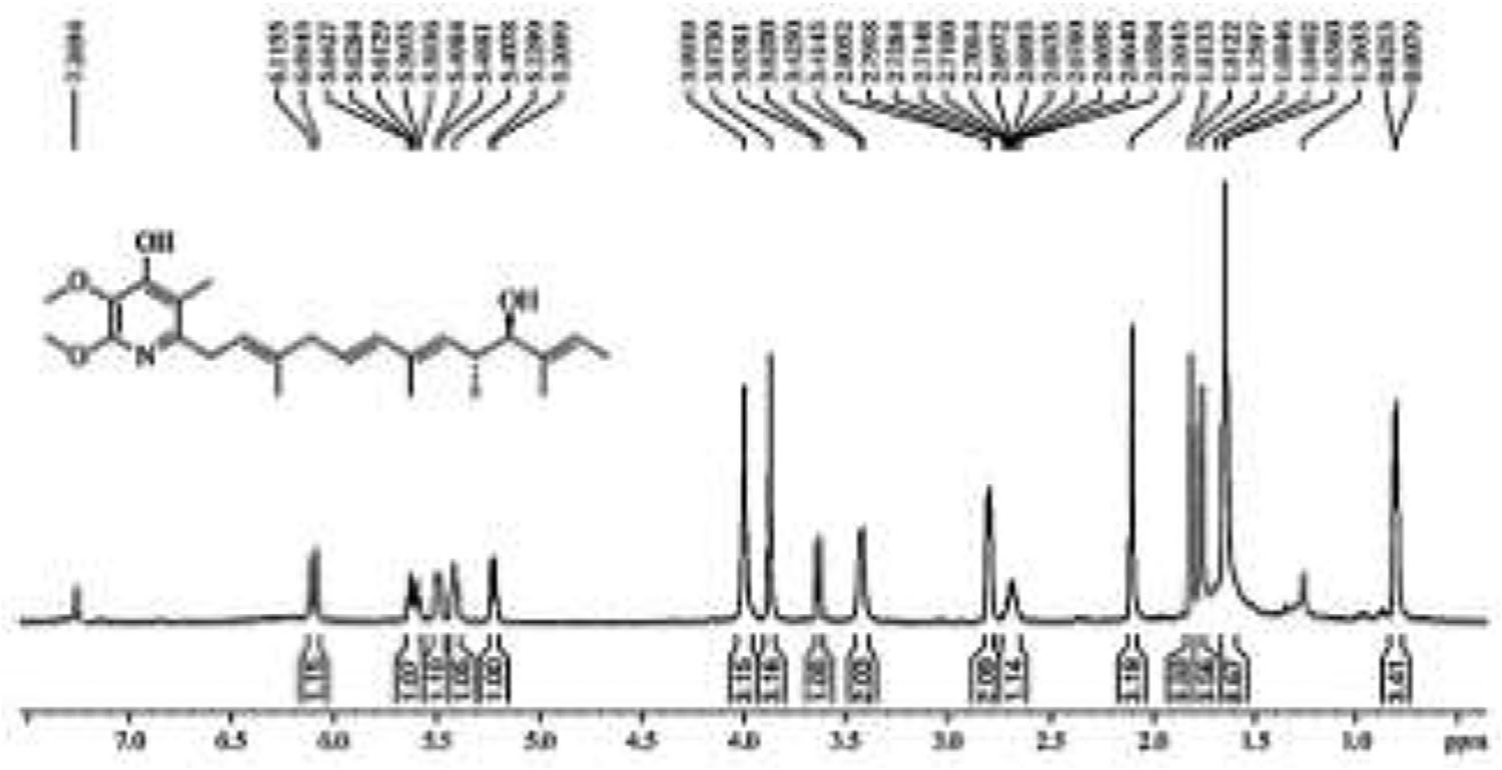
^1^H NMR spectrum of pieridicin A1 (**1**), in CDCI3. ChatGPT Plus 5.3 (accessed in February 2026) was used to enhance figure resolution. The final image was reviewed for accuracy by the authors.

The molecular formula C_25_H_37_NO_4_ (nine degrees of unsaturation) of **1** was determined by the high‐resolution mass spectrometry (HRESIMS) from the protonated molecular ion at *m/z* 416.2848 [M+H]^+^ and the ion at *m/z* 398.2560, which indicated the loss of a water molecule [M+H‐H_2_O]^+^ (Figure 2). Its ^1^H NMR spectrum (Figure 3) showed signals for olefinic protons at *δ*_H_ 6.10–5.20, two methylenes *δ*_H_ at 3.41 and 3.80; two methoxyl groups *δ*_H_ at 3.87 and 3.99, two methines at *δ*_H_ 3.62 and 2.70, and six methyl groups at *δ*_H_ at 2.10–0.80. Comparison of the NMR data of **1** with those of piericidin A1 showed a good match [30, 31, 32, 33]. The chromatogram of BRA‐035‐F7 (SI.5B) shows in the LC‐MS a peak with retention time (*t*_R_) at 24.50 min, whose MS exhibited a peak to the molecule protonated [M+H]^+^ at *m/z* 578.3431, indicating the molecular formula C_31_H_47_NO_9_ (10 degrees of unsaturation). The mass fragment at *m/z* 398.2710 (SI.7) indicated the loss of the glucose moiety [M+H‐Glu]^+^, leading us to suggest the structure of **2** as glucopiericidin A1 (**2**) [34, 35]. Another compound with *t*_R_ 26.8 min (SI.8) and molecular formula of C_25_H_37_NO_5_, deduced from the protonated molecular ion [M+H]^+^ with *m/z* 432.2808, was suggested to be piericidin C1 (**3**) [35, 36].

**FIGURE 4:**
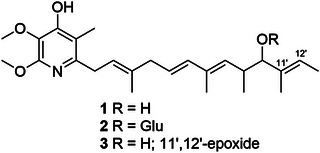
Chemical structure of piericidins isolated from the extract of strain *Streptomyces* sp. BRA‐035: Piericidin A (**1**), glucopiericidin A1 (**2**) and piericidin C1 (**3**).

Piericidins A and B were first isolated from *S. mobaraensis* in 1963 for insecticidal application [32]. Later, the antimicrobial and antitumor potential of piericidins, including **1** and **2**, was reported [34, 37, 38, 39]. From the 60's to the present, several isoforms of piericidins were discovered from natural sources, most of them obtained from marine and terrestrial bacteria of the genus *Streptomyces*, with a few exceptions occurring in other genera within *Actinomycetota phylum* [24]. To corroborate this information, here we describe the isolation and identification of three piericidins produced by a *Streptomyces* strain recovered from *P. variabilis*. The chemical structure of **1** consists of a 2,3‐dimethoxy‐5‐methyl‐4‐pyridinol ring containing at C‐6, an unsaturated poly‐methylated side chain [40] (Figure 4). The biosynthetic pathway of piericidin A1 was unraveled from the sequencing of the complete genome of *S. piomogeues* [33], which showed six modular polyketide synthases, two methyltransferases, two amidotransferases—one of which is an ATP‐dependent aminotransferase for the N atom—and a monooxygenase are required to produce the substance. Some authors suggest that other natural piericidin aglycones (i.e., piericidin C1) have apparent production during the fermentative growth process or extraction phases [30], while glycosidic derivatives are produced by glycosylation of piericidin A1 as a consequence of the long period of growth in culture medium [24, 34].

### 2.3 Effect of Piericidin A1 on the Viability of Cancer Cells In Vitro

The effects of **1** on the cell viability of seven tumor cell lines were investigated by MTT assay. Compound **1** showed high potency against OVCAR‐8, PC‐3/M, HCT‐116, SF‐295, and PC‐3 cell lines, with IC_50_ values ranging from 500 fM (OVCAR‐8) to 9.0 nM (PC‐3) (Tables 2 and SI.9). HL‐60 and B16‐F10 cell lines were resistant to **1** (IC_50_ >12 µM). Other studies have investigated the antiproliferative effect of piericidin A1 against tumor cell lines, revealing nanomolar‐ranged IC_50_ values overall; however there is a cell line‐dependent variability in biological activity. In fact, one study shows that while more sensitive cell lines, such as murine neuroblastoma cells (Neuro‐2a) displayed an IC_50_ of 0.21 nM, rat glioma cells (C6) remained unaffected by piericidin A1 (IC_50_ > 1000 nM) [41]. Likewise, human epidermal carcinoma (A431) cell lines were also reported to be resistant to exposure to the compound [42], whereas murine colon carcinoma (HT‐29) and fibrosarcoma (HT1080) cell lines showed piericidin A1 inhibits cell growth with IC_50_ values of 7.7 and 10.6 nM, respectively [43]. Furthermore, evaluation of piericidin A1 on L929 mouse fibroblasts revealed a high cytotoxic profile, with a recorded IC_50_ of 0.43 nM [44]. Such activity levels in a non‐tumorigenic cell line suggest that the cytotoxic mechanism of piericidin A1, while potent, may exhibit distinct sensitivity thresholds depending on the specific cellular model employed. Here, we demonstrate the effect of compound **1** in previously unreported cell lines, such as PC‐3 M (metastatic prostate carcinoma) and OVCAR‐8 (ovarian adenocarcinoma), with IC_50_ in the picomolar (1.8 pM) and femtomolar (500 fM) ranges, respectively (Table 2). Paclitaxel, a standard agent in ovarian cancer chemotherapy, has shown IC_50_ values in the low nanomolar range (0.4–3.4 nM) in ovarian carcinoma cell lines, such as OVCAR‐3, CAOV‐3, and SKOV‐3 in vitro [45]. In prostate cancer preclinical models, the first‐choice agent docetaxel exhibited IC_50_ values in the picomolar range (e.g., ∼0.6 nM in PC‐3 and ∼0.5 nM in DU145 cells), underscoring its high potency in vitro [46]. Collectively, these data indicate that the anticancer activity of compound 1 observed in this study has in vitro potency comparable to that of gold‐standard chemotherapeutic agents.

**TABLE 2:** Cytotoxic activity of **1** on a panel of tumor cell lines assessed by the MTT assay. Results are shown as the mean inhibitory concentration (IC_50_) and 95% confidence intervals (CI95%) on tumor cell lines after 72 h incubation. Data represent the mean of three independent experiments (*n* = 3), determined by nonlinear regression analysis using GraphPad Prism v.10.0.

Cell line	Tissue origin	Species	IC_50_ (nM)	CI95%
PC‐3	Prostate carcinoma	Human	9.084	2.491 – 33.13
PC‐3/M	Metastatic prostate carcinoma	Human	0.0018	0.0002 – 0.118
HL‐60	Acute promyelocytic leukemia	Human	>12000	—^a^
HCT‐116	Colorectal carcinoma	Human	0.144	0.062 – 0.338
SF‐295	Glioblastoma	Human	1.418	0.947 – 2.123
OVCAR‐8	Ovarian adenocarcinoma	Human	0.0005	0.0002 – 0.013
B16‐F10	Metastatic melanoma	Murine	>12000	—^a^

^a^ Not determined by nonlinear regression.

In this scenario, the unprecedented IC_50_ values obtained for piericidin A1 in the present study highlight the importance of screening programs and the need for further investigation of previously studied compounds. Piericidins present a 4‐hydroxypiridine nucleus (cyclic head) followed by a branched unsaturated side chain (hydrophobic tail), which is structurally correlated to ubiquinone Q_10_ (coenzyme Q), responsible for energy production through the mitochondrial respiratory chain [47, 48]. Indeed, piericidins are known to interfere with electron transport across the mitochondrial inner membrane in mammalian cells, specifically by blocking the first step of the transport chain, NADH‐ubiquinone oxidoreductase (complex I) [30, 31]. Due to their structural similarity and physiological effect, piericidins can be classified as coenzyme Q antagonists, along with other complex I inhibitors, such as stigmatellin and rotenone [49]. The consequences of blocking the mitochondrial respiratory chain with piericidins include reduced ATP production and increased ROS production. To a large extent, such energetic and reactive changes lead to cell death [50].

The process of tumorigenesis involves the acquisition of key features that confer adaptive advantages for tumor cell proliferation, survival, and dissemination [51]. It is worth noting that tumor cell metabolism must enable uninterrupted cellular proliferation, which implies a high energy demand to sustain these processes [52]. This is particularly valid for cells cultured in vitro, which are kept in exponential growth to enable cytotoxic studies. The ATP generation via oxidative phosphorylation is the most efficient pathway; however, glycolysis also plays an important role in cancer cells [53]. Glycolysis bypasses the oxidative phosphorylation pathway to overcome hypoxic conditions caused by rapid proliferation. Known as the Warburg effect, glycolytic metabolism suppresses the oxidative pathway in some cancer cells, even under aerobic conditions [54, 55, 56]. That being said, the contribution of mitochondrial respiration to energy production in malignant cells remains debated, and this pathway may represent a potential therapeutic target in cancer. In contrast to the widespread concept of glycolysis dependence, cancer cells rely on mitochondrial respiration to oxidize multiple metabolic fuels and produce most of their cellular energy. Furthermore, several mitochondrial respiratory chain subunits are critical for cancer cell growth, metastasis, and invasion [57]. The difference in sensitivity of the various cell lines assessed herein to **1** correlates with singularities in energy pathways. According to the literature, B16‐F10 is highly dependent on glycolysis and produces high amounts of lactate under aerobic conditions [58]. HL‐60 depicts high plasticity on energetic pathways, switching sources to adapt to different conditions [59]. Indeed, these cell lines displayed remarkable resistance to **1** (Table 2). On the other hand, PC‐3, PC‐3/M, OVCAR, SF295, and HCT‐116 are reported to have a greater dependence on oxidative phosphorylation to produce ATP [60, 61] and show greater susceptibility to **1**. This is a key point to explain the variation in sensitivity and, therefore, in the IC_50_ values obtained for **1** in the different cell lines and deserves further investigation.

### 2.4 Cell Count and Membrane Integrity of Piericidin A1 on Tumor Cells

It must be acknowledged that the blockage in the electron transport chain induced by compound **1** can impact the cell viability measurements of piericidin A1‐treated cells carried out by the MTT assay. The MTT assay is a widely used colorimetric method in drug screening programs. It provides a convenient and rapid approach to indirectly assess cell viability through their cellular metabolic activity [62]. In this assay, MTT (3‐(4,5‐dimethylthiazol‐2‐yl)‐2,5‐diphenyltetrazolium bromide), a yellow tetrazolium salt, is reduced to insoluble purple crystals of formazan. Quantification is performed by measuring solubilized formazan spectrophotometrically [63]. The reduction mechanism involves cleavage of the tetrazolium ring by dehydrogenase enzymes and depends on the cell's metabolic activity driven by mitochondrial and cytosolic NADH flux [64]. Therefore, accepting that compound **1** interferes with cellular metabolism, we hypothesized that this event may result in under‐conversion of MTT to formazan, possibly caused by perturbations in oxidoreductase reactions [65].

**FIGURE 5:**
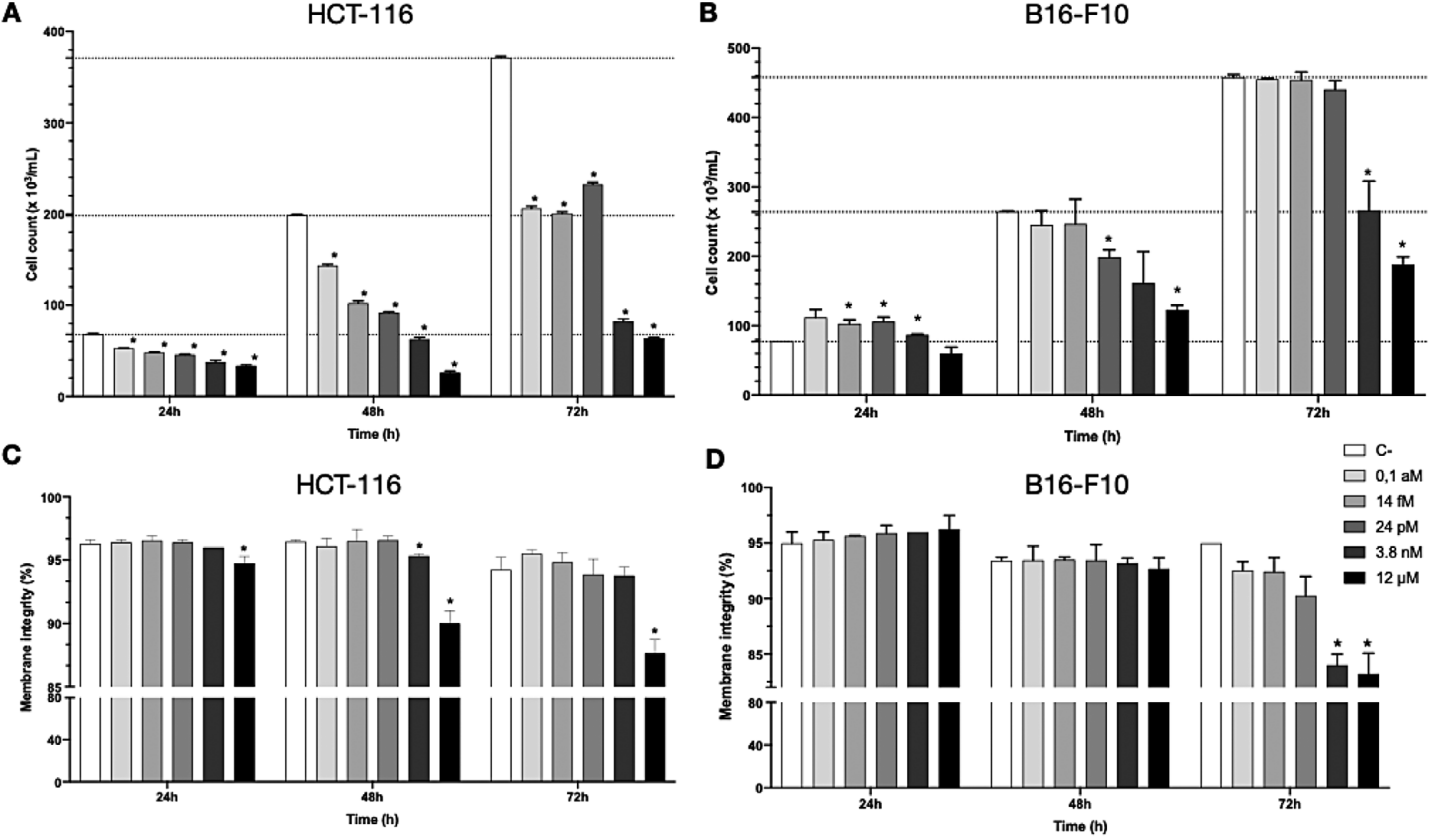
Effect of compound **1** on cell count and viability of HCT‐116 and B16‐F10 cell lines under different conditions. (A) Cell count of HCT‐116 after 24, 48, and 72 h incubation. (B) Cell count of B16‐F10 after 24, 48, and 72 h incubation. (C) Cell viability of HCT‐116 after 24, 48, and 72 h incubation. (D) Cell viability of B16‐F10 after 24, 48, and 72 h incubation. Data represent the mean±SEM of three independent experiments (*n* = 3). Statistical differences were analyzed using one‐way ANOVA followed by Dunnett's post‐hoc test using GraphPad Prism v.10.0. * *p* < 0.05.

To alternatively address the cytotoxicity profile of compound **1**, we resorted to a direct cell counting/cell viability method. Using flow cytometry to evaluate the effects of **1** on cell number and membrane integrity (cell death) after 24, 48, and 72 h exposure, we selected one among the piericidin A1‐sensitive (HCT‐116) and resistant (B16‐F10) cell lines from the panel initially assessed herein. HCT‐116 cells incubated with **1** showed significantly diminished (*p* < 0.05) cell counts compared to the negative control (C‐) at 24, 48, and 72 h on concentrations ranging from 0.1aM to 12µM (Figure 5A). At 72 h, cultures of HCT‐116 cells exposed to 0.1 aM–24 pM of **1** were reduced to nearly 50% of the cell count of the control, while at 3.8 nM–12 µM, that number came down to 25%. However, no change in HCT‐116 cell viability was detected in most treatment conditions, except for at 12 µM treatment after both 48 and 72 h (Figure 5C). B16‐F10, in turn, showed decreased cell counts at 3.8 nM and 12 µM after 48 and 72 h incubation, reaching between 50% and 40% cell counts after 72 h exposure to **1**, respectively (Figure 5B). A decrease in membrane integrity of these cells was observed for the same concentrations and corresponding time periods (Figure 5D). These results indicate that, in fact, there is a marked difference in sensitivity to **1** between HCT‐116 and B16‐F10 cell lines and, furthermore, that the MTT assay may underestimate the cytotoxicity of compounds that promote mitochondrial uncoupling.

The pharmaceutical agent metformin, although widely prescribed for type 2 diabetes, has been investigated for potential anticancer effects due to its ability to inhibit mitochondrial respiratory chain and alter cellular metabolism [66]. As observed for compound **1**, the role of metformin in the inhibition of tumor growth is also related to the impairment of mitochondrial complex I in HCT‐116 cells [67]. The blockage of complex I is crucial for metformin's anticancer activity, where cells decrease their proliferation rate under glucose‐supplied conditions; however, no change in cell viability is observed. Additionally, depriving cells of glucose significantly increases cell death [67]. Similarly, our experiments show that HCT‐116 cells exposed to compound **1** decrease cell proliferation at concentrations as low as 0.1 aM under typical glucose availability while maintaining cell viability (Figure 5A,B). According to other studies, the disruption of energy supply caused by the blockage of mitochondrial complex I is sufficient to stop or reduce cell proliferation in the presence of glucose; however, cells remain active with basal metabolic activity due to energy generation through the glycolytic pathway [55, 67, 68]. Furthermore, a reduction in cell proliferation rates, particularly in the HCT‐116 cell line, supports the MTT assay results despite possible disturbances in MTT reduction reactions and, therefore, an underestimation of IC_50_ values.

Targeting mitochondrial metabolism to induce energy stress in tumor cells has long been explored as an anticancer strategy, yet no mitochondrial inhibitor has been successfully developed for this purpose [25, 69]. A major challenge lies in inhibiting metabolic pathways that are also essential in healthy cells, thereby narrowing the therapeutic window and increasing the risk of systemic toxicity [70]. Selective inhibitors of complex I, such as piericidin and metformin, disrupt cellular energy homeostasis and limit the biosynthesis of nucleic acid precursors and macromolecules required for proliferation, thereby suppressing tumor growth [71]. In addition to metformin, other Food and Drug Administration‐approved drugs, such as canagliflozin, also exhibit anticancer activity through complex I inhibition [72]. However, these agents have not been repurposed clinically, largely due to insufficient efficacy at tolerable doses [70, 71]. To overcome the limitations of mitochondrial blockers in anticancer therapies, targeted delivery strategies have emerged as a promising approach. Cancer cells exhibit a markedly elevated mitochondrial membrane potential, which can be exploited to enhance the selective accumulation of small‐molecule inhibitors of mitochondrial energy metabolism. Conjugation of these compounds to the lipophilic cation triphenylphosphonium (TPP^+^) promotes preferential mitochondrial accumulation driven by the negative mitochondrial membrane potential (*ΔΨ*m), which electrophoretically attracts and concentrates TPP^+^‐linked molecules across the inner mitochondrial membrane. Because tumor cells often display hyperpolarized mitochondria, this strategy enables the use of this feature to selectively target cancer cells [73]. This strategy is further supported by studies with a metformin–TPP^+^ derivative, which exhibits up to 1000‐fold greater inhibition of complex I and antiproliferative activity in pancreatic cancer cell models [74], highlighting a compelling avenue for developing next‐generation anticancer therapeutics. Further studies should explore the translational potential of piericidins as mitochondrial complex I inhibitors, particularly in the context of their application as pharmacological tools and tumor‐sensitizing agents. Evaluating their performance in combination with standard chemotherapeutics, as well as their safety, selectivity, and suitability for targeted delivery strategies in preclinical models, will be essential to sustain their promise as candidates for anticancer drug development.

## 3 Conclusions

*P. variabilis* from Brazilian tropical waters harbors bacteria capable of producing bioactive metabolites. Herein, piericidin A1 (**1**), glucopiericidin A1 (**2**), and piericidin C1 (**3**) were isolated from *Streptomyces* sp. BRA‐035, an Actinomycetota strain associated with *P. variabilis*. Compound **1** exhibits a highly potent antiproliferative activity at sub‐femtomolar concentrations against various cancer cell lines, especially toward cells that favor an oxidative metabolism for energy supply. These findings reinforce the relevance of cnidarian‐associated microbiota as a promising source of bioactive natural products.

## 4 Experimental

### 4.1 Sampling of Zoantharians

Colonies of *P. variabilis* were sampled during low tide at Taíba and Paracuru beaches in tide pools of beachrocks (3°30'20.46“ S, 38°53'54.31” W and 3°39′5″ S, 39°01′3″ W, respectively), located in the state of Ceará on the tropical northeast coast of Brazil, using surgical forceps and a stainless‐steel spatula rinsed with 70% ethanol. Polyps were rinsed with 70% ethanol following a wash in sterile seawater. Sampled material was stored in sterile sample bags, packed in styrofoam boxes cooled with icepacks, and taken for laboratory processing within 3 h. Authorization and Information System (SISBIO number 48522‐2, SisGen number AC0781C).

### 4.2 Isolation of Bacterial Strains Associated With Zoantharians

The colonies of zoantharians sampled were processed under sterile conditions as follows: samples were cut extensively, heated to 56°C for 15 min, spread on agar‐media plates, and incubated at room temperature (approximately 25°C) for up to 8 weeks. After 2 to 12 weeks of incubation, 10 strains have grown and were isolated. For cultivation of bacteria associated with *P. variabilis*, the following media were used: SWA (Sea Water Agar—18 g of agar; 1 L of filtered seawater diluted 75% in dH_2_0), TM (Trace Minerals Agar–0.1 g/L glucose; 0.1 g/L yeast extract; 0.5 g/L K_2_HPO_4_; 0.7 g/L Na_2_HPO_4_; 0.1 g/L KNO_3_; 0.3 g/L NaCl; 0.1 g/L MgSO_4_.7H_2_O; 0.02 g/L, CaCl_2_.2H_2_0; 18 g/L agar; 1L seawater diluted 75% in dH_2_0) and SCA (Starch‐Casein Agar—10 g/L soluble starch; 1 g/L casein powder; 37 g/L seawater preparation; 15 g/L agar; 1 L dH_2_O). Isolation of strains was carried out using A1 agar media (10 g/L soluble starch; 4 g/L yeast extract; 2 g/L peptone; 18 g/L agar; 1 L filtered seawater diluted 75% in dH_2_0). Purified bacterial strains were grown in liquid A1 broth to produce the crude extract, deposited in a bacterial bank (MicroMarin) at −80°C in 25%, and glycerol and for genetic extraction for molecular identification. Isolated strains were cultivated for 8 days (26 ±1°C, under constant agitation) and extracted with AcOEt (Synth) (1:1, during 2 h under agitation). Solvent was removed by reduced‐pressure rotary evaporation.

### 4.3 BRA‐035 Molecular Identification by 16S rRNA Sequencing

Genomic DNA from strain BRA‐035 was extracted using the DNeasy Blood & Tissue Kit (QIAGEN, Germany) following the manufacturer's instructions. PCR amplification of the 16S rRNA gene was performed using the Illustra Ready‐To‐Go RT‐PCR Beads kit (GE Healthcare Life Sciences, USA) and the universal primers F27 and R1492. PCR products were visualized by agarose gel electrophoresis, quantified using a NanoDrop spectrophotometer (NanoDrop 2000c/2000 UV–Vis, Thermo Scientific, USA), and purified with the MiniElute PCR Purification Kit (QIAGEN, Germany). Sequencing was carried out by the Sanger method at Macrogen Inc. (Republic of Korea). The chromatograms were trimmed, and assembled, and the consensus sequence was generated in Geneious Prime (Biomatters Ltd., New Zealand). Molecular identification was performed through comparative analysis of the BRA‐035 16S rRNA gene sequence using the BLAST (Basic Local Alignment Search Tool) database hosted by NCBI. Closely related sequences were retrieved from the EzBioCloud platform (https://www.ezbiocloud.net) and aligned in UGENE (Unipro, Russia); the *Micromonospora carbonacea* sequence (GenBank accession NR_037043) was included as the outgroup for phylogenetic analyses. The multiple sequence alignment file was then used to construct a phylogenetic tree using the RAxML‐HPC BlackBox tool available through the CIPRES Science Gateway platform (San Diego Supercomputer Center, USA). The resulting tree was visualized and edited in iTOL version 6.9.0 (Interactive Tree of Life; Letunic and Bork, 2021). The BRA_035 sequence was submitted to the NCBI GenBank platform database and can be found under the code PX514930.

### 4.4 Bioassay‐Guided Fractionation and Isolation of Compound 1

*Streptomyces*‐BRA035 culture broth (10 L) yields 150 mg of extract (EAcE‐035). The fractionation direct injection was performed by HPLC using a Shimadzu UFLC system equipped with SPD‐M20A diode array UV–vis detector. Separations were performed using a Phenomenex reversed‐phase column (250×4.6 mm, i.d. 5 µm). The solvents used for the analyses consisted of water (solvent A) and acetonitrile (solvent B) transported at 35°C, with an injection volume (loop) of 200 µL and a flow rate of 4.72 mL/min (5%–95% A 0–30 min and 100% B 30–40 min). The scanning wavelength range of the PDA was set at 190–400 nm, and chromatograms were recorded between 210 and 400 nm. Results from seven fractions (F1‐P8) were tested for cytotoxicity to guide the isolation of the active substance. The identification of piericidin A1 (PA1) (F8; 1.0 mg) and derivatives present in fraction F7 (0.5 mg) was performed through the interpretation of high‐resolution electrospray ionization mass spectra (HRESIMS) acquired using a liquid chromatography‐mass spectrometry ion trap and time‐of‐flight (LCMS‐IT‐TOF, Shimadzu) spectrometer, consisting of a UFLC (ultra‐fast liquid chromatography) system coupled to an IT‐TOF mass spectrometer equipped with an electrospray ionization (ESI) source operating either in positive or negative mode. The mass spectra were recorded in the range of *m/z* 100–1000 Da, using a potential of 4.0 kV on the capillary and nitrogen as the desolvation gas. Chromatographic runs were performed on a BEH C18 column (150 mm × 2.1 mm, 1.7 µm) at 40°C, with 2 µL of sample injected at 20°C. The mobile phase is composed of 0.1% formic acid in water (A) and 0.1% formic acid in acetonitrile (B) at a flow rate of 0.4 mL/min (gradient system 5%–95% A 0–30 min and 100% B 30–40 min). As well, the nuclear magnetic resonance (NMR) spectrum of hydrogen (^1^H) was obtained on a Bruker Avance DRX‐500 (500 MHz to ^1^H). Chemical shifts are given relative to CDCl_3_ at 7.27 ppm.

### 4.5 Cell Culture and MTT Assay

Crude extracts, bioguided‐fractionation, and **1** were tested against PC‐3/M (metastatic prostate carcinoma, NCI‐DTP, RRID:CVCL_9555) cell lineage, while **1** cytotoxicity potential was also evaluated against PC‐3 (prostate carcinoma, ATCC Cat# CRL‐1435 RRID:CVCL_0035), HL‐60 (promyelocytic leukemia, ATCC Cat# CCL‐240, RRID:CVCL_0002), OVCAR‐8 (ovarian carcinoma, NCI‐DTP, RRID:CVCL_1629), HCT‐116 (colorectal carcinoma, ATCC Cat# CCL‐247, RRID:CVCL_0291), SF‐295 (glioblastoma, NCI‐DPT, RRID:CVCL_1690), and B16‐F10 (murine melanoma, ATCC Cat# CRL‐6475, RRID:CVCL_0159) cell lines. Lineages were acquired by the American Type Culture Collection (ATCC, Manassas, VA, USA) and National Cancer Institute (NCI, Bethesda, MD, USA), and grown in RPMI‐1640 (Thermo Fisher Scientific, Waltham, MA, USA) medium supplemented with 10% fetal bovine serum, 2 mM glutamine, 1000 U/mL streptomycin, and 100 µg/mL penicillin (Sigma‐Aldrich Co.) in a controlled atmosphere of 5% CO_2_ at 37°C. For MTT assay, cells were seeded into 96‐well plates at 5 × 10^4^ cell/mL 24 h prior to the addition of samples. Extracts, fractions, and **1** were diluted in filtered‐sterile Dimethyl Sulfoxide (DMSO—Synth). Control groups received the same amount of DMSO, and Doxorubicin (Sigma‐Aldrich Co.) (0.01 to 5.0 µg/mL) was used as positive control. With 3 h remaining to complete the incubation time for each experiment, the medium was replaced with fresh medium containing 0.5 µg/mL of MTT (Sigma‐Aldrich Co.) and incubated again to fulfill 72 h. Media were removed, and dried formazan crystals were diluted in 150 µL of DMSO. A plate spectrometer reader (DTX 880 Multimode Detector, Beckman Coulter, Inc. Fullerton, CA, USA) was used to measure absorbance at 570 nm. IC_50_ and CI95 parameters were determined by nonlinear regression using GraphPad Prism v10.0. Crude extracts screening concentration was 50 µg/mL. For dose‐response investigations, BRA‐035 crude extract ranged from 0.0031 to 50.0 µg/mL, BRA‐035 large‐scale crude extract ranged from 0.00005 to 10.0 µg/mL, bioguided‐fractions ranged from 50 to 0.016 µg/mL, F7 ranged from 1 to 1.02^E‐07^ µg/mL, **1** ranged from 1.01^E‐15^ 12 µM in PC‐3, PC‐3/M, OVCAR‐8, HCT‐116, SF‐295, B16‐F10, and from 5.74^E‐06^ 12 µg/mL in HL‐60.

### 4.6 Cell Counting and Viability by Flow Cytometry

HCT‐116 and B16‐F10 cells were seeded in 24‐well plates, treated with **1** at 0.1 aM, 14 fM, 24 pM, 3.8 nM, and 12 µM concentration, and incubated for 24, 48, and 72 h. After incubation, cells were trypsinized, resuspended in 500 µL of media, and centrifuged at 1500 rpm for 2 min (Hettich, model Universal 320R). Pellets were resuspended in 250 µL of a 5 µg/mL PI solution (Sigma Aldrich Co.) diluted in phosphate‐buffered saline (PBS). After 15 min of incubation, samples were washed with PBS to remove excess PI and analyzed on a BD Accuri C6 flow cytometer (BD Bioscience). 10,000 events were counted from each sample, excluding debris and doublets. The cells were arranged according to their linear dispersion, volume, and granularity. Percentages of cells with intact or disrupted plasma membranes were analyzed using markers to delimit the cell population at regions with the lowest and highest fluorescence, compared with the negative control, using the BD Accuri TM C6 Software v.1.0.264.21. Data were analyzed using the mean and corresponding standard errors from three independent experiments in GraphPad Software v10.0. Significant differences between treatment groups were assessed using analysis of variance (ANOVA), followed by Dunnett's test at the 5% significance level (*p* < 0.05).

### 4.7 Statistical Analysis

All data are expressed as the mean ± standard error of the mean (SEM) or as mean values with their respective 95% confidence intervals (CI 95%), derived from at least three independent experiments (*n* = 3). The half‐maximal inhibitory concentration (IC_50_) and CI95% were determined through nonlinear regression analysis of the dose–response curves. For the initial screening of crude extracts and flow cytometry assays, statistical significance was assessed using one‐way analysis of variance (ANOVA) followed by Dunnett's post‐hoc test for multiple comparisons against the negative control. For time‐dependent assays (24, 48, and 72 h), two‐way ANOVA was applied. A *p*‐value < 0.05 was considered statistically significant. All statistical analyses and graphical representations were performed using GraphPad Prism software version 10.0 (GraphPad Software, San Diego, CA, USA). Flow cytometry data were processed using the BD Accuri C6 Software v.1.0.264.21.

## Author Contributions

**Bianca Del B. Sahm**: conceptualization, investigation, data curation, formal analysis, original draft writing, writing – review and editing. **Katharine G. D. Florêncio**: investigation, data curation, formal analysis, original draft writing, writing – review and editing. **Francisco das Chagas L. Pinto**: investigation, data curation, formal analysis, original draft writing. **Ana I. V. Maia**: investigation. **Carlos A. M. Rocha**: investigation. **Paula C. Jimenez**: investigation, formal analysis, writing – review and editing. **Otília D. L. Pessoa**: investigation, data curation. **Tito M. C. Lotufo**: data curation, writing – review. **Leticia V. Costa‐Lotufo**: conceptualization, project administration, funding acquisition, resources, writing – review. **Diego V. Wilke**: conceptualization, investigation, data curation, project administration, funding acquisition, resources, original draft writing, writing – review and editing.

## Conflicts of Interest

The authors declare no conflicts of interest.

## Supplementary Material

Supporting information for this article is available on the **WWW** under https://doi.org/10.1002/cbdv.202503815.

## Supporting information




**Supporting file**: cbdv71237‐sup‐0001‐SuppMat

## Data Availability

The data that support the findings of this study are available in the supplementary material of this article.
