# Supplementary material for: Anticancer Potential of Piericidin A1 and Derivatives Isolated From Streptomyces sp. Associated With Palythoa variabilis From Brazilian Reefs
Source: Chem Biodivers. 2026 Apr 20;23:e03815. doi: 10.1002/cbdv.202503815 (PMC13095201; doi:10.1002/cbdv.202503815)
Supplement: Supplementary file 1 — Supporting file: cbdv71237‐sup‐0001‐SuppMat [file CBDV-23-e03815-s001.docx]

**Anticancer Potential of Piericidin A1 and Derivatives Isolated from *Streptomyces* sp. Associated with *Palythoa variabilis* from Brazilian Reefs**

Bianca Del B. Sahm^
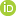
a^, Katharine G. D. Florêncio^
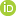
b^, Francisco C. L. Pinto^
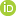
c^, Ana I. V. Maia^c^, Carlos A. M. Rocha^d^, Paula C. Jimenez^
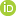
e^, Otília D. L. Pessoa^
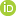
c^, Tito M. C. Lotufo^
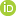
f^, Leticia V. Costa-Lotufo^
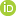
a^, and Diego V. Wilke^
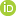
^*^,b^

^a^ Departamento de Farmacologia, Universidade de São Paulo, São Paulo, SP, Brazil

^b^ Núcleo de Pesquisa e Desenvolvimento de Medicamentos, Departamento de Fisiologia e Farmacologia, Universidade Federal do Ceará, Fortaleza, CE, Brazil (e-mail: [diegowilke@ufc.br](mailto:diegowilke@ufc.br))

^d^ Coordenação de Recursos Pesqueiros e Agronegócio, Instituto Federal de Educação, Ciência e Tecnologia do Pará, Belém, PA, Brazil

^c^ Departamento de Química Orgânica e Inorgânica, Universidade Federal do Ceará, Fortaleza, CE, Brazil

^e^ Instituto do Mar, Universidade Federal de São Paulo, Santos, SP, Brazil

^f^ Instituto Oceanográfico, Universidade de São Paulo, São Paulo, SP, Brazil

**Supporting Information**

**Table of contents**

[**SI.1.** Bacteria recovered from *Palythoa variabilis* and respective isolation media. 2](#_Toc226104383)

[**SI.2.** HPLC chromatogram of the crude extract obtained from strain *Streptomyces* sp. BRA-035. Red bars show the delimitation of fractions (numbered) collected at the determined retention times. Chat-GPT Plus 5.3 (accessed in February 2026) was used to enhance figure resolution. The final image was reviewed for accuracy by the authors. 3](#_Toc226104385)

[**SI.3.** Bioassay-guided fractionation of *Streptomyces* sp. BRA-035 crude extract. **A**, Cytotoxicity profile of BRA-035 large-scale crude extract against PC-3/M cell line after 72h of incubation assessed by the MTT assay. **B**, Cytotoxicity of HPLC-derived fractions against PC-3/M cell line after 72h of incubation assessed by the MTT assay. Data represent the mean and error bars represent 土SEM of three independent experiments (n=3), determined by non-linear regression analysis using GraphPad Prism v.10.0. 3](#_Toc226104386)

[**SI.4.** Cytotoxicity profile of fractions F7 (**A**) and F8 (**B**) against PC-3/M cell line after 72h of incubation assessed by the MTT assay. Data represent the mean and error bars represent 土SEM of three independent experiments (n=3), determined by non-linear regression analysis using GraphPad Prism v.10.0. 3](#_Toc226104387)

[**SI.5.** LCMS-IT-TOF chromatogram PDA at 215-400 nm of the fractions BRA035-F7 (**A**) and BRA035-F8 (**B**). 4](#_Toc226104388)

[**SI.6.** BRA035-F7 and BRA035-F8 LCMS-IT-TOF chromatogram data. 4](#_Toc226104389)

[**SI.7.** HRESIMS of glucopiericidin A1 (**2**). Chat-GPT Plus 5.3 (accessed in February 2026) was used to enhance figure resolution. The final image was reviewed for accuracy by the authors. 5](#_Toc226104390)

[**SI.8.** HRESIMS ofr piericidin C1 (**3**). Chat-GPT Plus 5.3 (accessed February 2026) was used to enhance figure resolution. Final image was reviewed for accuracy by authors. 5](#_Toc226104391)

[**SI.9.** Cytotoxicity profile of piericidin A1 (**1**) against different cell lines after 72h of incubation assessed by the MTT assay. Data represent the mean and error bars represent 土SEM of three independent experiments (n=3), determined by non-linear regression analysis using GraphPad Prism v.10.0. 6](#_Toc226104392)

#

# **SI.1.** Bacteria recovered from *Palythoa variabilis* and respective isolation media.

| **Strain** | **Isolation culture media** |
| --- | --- |
| BRA-035 | SCA |
| BRA-036 | SCA |
| BRA-045 | SCA |
| BRA-046 | SCA |
| BRA-060 | SWA |
| BRA-061 | SCA |

# ****

# **SI.2.** HPLC chromatogram of the crude extract obtained from strain *Streptomyces* sp. BRA-035. Red bars show the delimitation of fractions (numbered) collected at the determined retention times. Chat-GPT Plus 5.3 (accessed in February 2026) was used to enhance figure resolution. The final image was reviewed for accuracy by the authors.

# **SI.3.** Bioassay-guided fractionation of *Streptomyces* sp. BRA-035 crude extract. **A**, Cytotoxicity profile of BRA-035 large-scale crude extract against PC-3/M cell line after 72h of incubation assessed by the MTT assay. **B**, Cytotoxicity of HPLC-derived fractions against PC-3/M cell line after 72h of incubation assessed by the MTT assay. Data represent the mean and error bars represent 土SEM of three independent experiments (n=3), determined by non-linear regression analysis using GraphPad Prism v.10.0.

# **SI.4.** Cytotoxicity profile of fractions F7 (**A**) and F8 (**B**) against PC-3/M cell line after 72h of incubation assessed by the MTT assay. Data represent the mean and error bars represent 土SEM of three independent experiments (n=3), determined by non-linear regression analysis using GraphPad Prism v.10.0.

# **SI.5.** LCMS-IT-TOF chromatogram PDA at 215-400 nm of the fractions BRA035-F7 (**A**) and BRA035-F8 (**B**).

# **SI.6.** BRA035-F7 and BRA035-F8 LCMS-IT-TOF chromatogram data.

| **Sample** | **Peak #** | **Retention time (min)** | **Area** | **Height** | **Area %** | **Height %** |
| --- | --- | --- | --- | --- | --- | --- |
| BRA-035-F7^a^ | 1 | 1.868 | 1660716 | 435730 | 8.312 | 30.148 |
|  | 2 | 18.070 | 5968958 | 33018 | 29.876 | 2.284 |
|  | 3 | 20.768 | 1270632 | 67754 | 6.360 | 4.688 |
|  | 4 | 22.967 | 567464 | 19377 | 2.840 | 1.341 |
|  | 5 | 24.960 | 542973 | 33756 | 2.718 | 2.336 |
|  | 6 | 26.828 | 1868159 | 201431 | 9.351 | 13.937 |
|  | 7 | 28.940 | 346169 | 19486 | 1.733 | 1.348 |
|  | 8 | 26.828 | 4251490 | 445478 | 21.280 | 30.822 |
|  | 9 | 28.940 | 2553391 | 73483 | 12.781 | 5.084 |
|  | 10 | 30.434 | 948823 | 115800 | 4.749 | 8.012 |
| Total BRA-035-F7 | - | - | 19978775 | 1445313 | 100.000 | 100.000 |
| BRA-035-F8^b^ | 1 | 1.872 | 2576641 | 646406 | 4.950 | 13.795 |
|  | 2 | 7.682 | 1723818 | 64872 | 3.312 | 1.384 |
|  | 3 | 30.304 | 47748492 | 3974983 | 91.738 | 84.822 |
| Total BRA-035-F8 | - | - | 52048951 | 4686261 | 100.000 | 100.000 |
| ^a^ PDA Ch1 210nm - 400nm 4nm . ^b^ PDA Ch1 215nm - 400nm 4nm. | | | | |  |  |

#

# **SI.7.** HRESIMS of glucopiericidin A1 (**2**). Chat-GPT Plus 5.3 (accessed in February 2026) was used to enhance figure resolution. The final image was reviewed for accuracy by the authors.

# **SI.8.** HRESIMS ofr piericidin C1 (**3**). Chat-GPT Plus 5.3 (accessed February 2026) was used to enhance figure resolution. Final image was reviewed for accuracy by authors.

# **SI.9.** Cytotoxicity profile of piericidin A1 (**1**) against different cell lines after 72h of incubation assessed by the MTT assay. Data represent the mean and error bars represent 土SEM of three independent experiments (n=3), determined by non-linear regression analysis using GraphPad Prism v.10.0.
